# Background Subtraction Approach based on Independent Component Analysis

**DOI:** 10.3390/s100606092

**Published:** 2010-06-18

**Authors:** Hugo Jiménez-Hernández

**Affiliations:** Centro de Investigación en Ciencia Aplicada y Tecnología Aplicada Cerro Blanco No. 141. Col. Colinas del Cimatario, Santiago de Querétaro, Querétaro, Mexico

**Keywords:** background subtraction, independent component analysis, motion detection

## Abstract

In this work, a new approach to background subtraction based on independent component analysis is presented. This approach assumes that background and foreground information are mixed in a given sequence of images. Then, foreground and background components are identified, if their probability density functions are separable from a mixed space. Afterwards, the components estimation process consists in calculating an unmixed matrix. The estimation of an unmixed matrix is based on a fast ICA algorithm, which is estimated as a Newton-Raphson maximization approach. Next, the motion components are represented by the mid-significant eigenvalues from the unmixed matrix. Finally, the results show the approach capabilities to detect efficiently motion in outdoors and indoors scenarios. The results show that the approach is robust to luminance conditions changes at scene.

## 1. Introduction

One of fundamental steps, in several computer vision systems, is the motion detection process. The moving objects represent the main features used to analyze the motion dynamics at scene. The background and foreground commonly represent fixed areas and moving areas respectively. The process of identifying foreground and background objects is a tough task. The background subtraction approach consists on label moving objects and fixed regions. However, there are several factors, like luminance, reflections, shadows, or even camera shaking that make this process difficult [1, 2].

The foreground labeling process can be considered as a general classification problem, where using a model *M*, we try to estimate a set of parameters *P* = {*p*_1_, *p*_2_, . . .}, which correctly label/classify background and foreground objects. The parameter estimation uses previous knowledge about scenario and object properties. The capabilities of classification depend on both the raw data and the model *M*. It is usually pretended to build a single binary classifier [2–4] (if it only identifies fixed and moving zones) or multiple classifiers [1, 5] (if it wishes to model several types of moving objects and fixed layers). The success of motion detection for a particular model depends on the scene constraints, the data dynamics on both temporal and spatial conditions, and the separability of data under the current model *M*. In the literature, one of the most accepted approaches is the work of Stauffer and Grimson [3]. They proposed an approach based on the assumption that the foreground and the background can be modeled as a mixture of Gaussians (MOG), where the Gaussians with low probability represent moving objects. This approach is computationally efficient; moreover, the convergence velocity and the spatial object consistency are not considered. Furthermore, in their work, Toyama *et al.* [1] proposed a background approach capable of discarding periodic movements in background as the waves of the sea or the up and down of electric stairs. Moreover, this approach is limited to scenarios with the fixed luminance conditions. Next, Elgammal *et al.* [6] proposed an approach based on a pixel probability density function (pdf) approximation using last immediate frames, which are smoothed with a Gaussian kernel. Also, Horprasert *et al.* [2] proposed a method based on object segmentation that supports shadows and changing luminance conditions, against an increase of the computational complexity. The same form, Oliver *et al.* [7] proposed a background approach based on a temporal correlation of pixel values. Next, the Principal Component Analysis (PCA) [8] is applied to eliminate those components, which do not provide information to the model. This approach was improved to support small luminance changes by Rymel *et al.* [9]. More recently, Han *et al.* [10] presented an approach to estimate a set of models of pixel intensities based on a combination of mean-shift pdf’s estimators and a propagated Gaussian kernel. Following the trend, Tang and Miao [11] presented an extension to the MOG approach, which supports shadows. Other relevant work is proposed by Zhou *et al.* [12], where they show a different background approach, based on the analysis of image texture using Gabor filters. Finally, at a recent time, in their work [13], Du-Ming and Shiah-Chin proposed a way to detect motion based on Independent Component Analysis (ICA), which is used to estimate the background information. This approach is based on the Particle Swarm Optimization (PSO) algorithm to search the best unmixing matrix. The background process consists on estimate the unmixing matrix using a background estimation and the current image. Afterwards, the approach, for consecutive frames, separates the background and the foreground objects as a single threshold classification task, using the same estimated unmixed matrix. Moreover, this approach is limited to scenarios where the background dependences between foreground and background never change. As a consequence, in outdoor scenarios this approach is not suitable.

This work presents a novel background subtraction approach based on Independent Component Analysis [14, 15]. This approach exploits the property of separability of the pdf in several components; in which one of them represents the background, and the rest of the components represent foreground areas and noise effects. This approach considers the task as a cocktail problem, where background and foreground information are mixed spatially and temporally. The process consists on identifying the pdf of the background from the rest, separating the different components as a linear independent component. The separation process is performed with an unmixing matrix. The parameters of the unmixing matrix are estimated as a maximization of non-Gaussian problem, which is solved via Newton-Raphson[16]. Background and foreground zones are detected by extracting the most and middle significant components from the unmixed matrix. To compensate the dynamic changes of the scenario, the approach is continuously estimating the unmixed matrix and the estimation of background information. As a consequence, this approach is adaptable to different luminance conditions. The paper is organized as follows. In §2, a background model based on ICA is presented. The parameter estimation for the unmixing matrix is discussed in §3. Next, the motion detection from the unmixed matrix is presented in §4. Afterwards, in §5 the approach is tested, comparing foreground detection with the MOG approach [3]. Both approaches are tested in several image sequences took from a PETS database [17, 18] and outdoors/indoors scenarios (gardens and vehicular intersections). Using the PETS database we can develop a background bench scheme to quantify the accuracy of tested approaches. Finally, the conclusion is shown.

## 2. Background Model

Given a set of consecutive images ϒ = {*I*_1_, *I*_2_, . . . , *I_n_*}, the information of moving objects and fixed objects are mixed. Each particular image pixel position **x** = [*x*_1_,*x*_2_] is indexed as *I_i_*(**x**) for *k* × *l* image dimensions. To start with, an *E*(ϒ) operator is defined. This operator mixes the images in ϒ and estimates the dominant color pixel value for each position **x**. Table 1 shows some common operators used for this purpose, even though, there are many others operators; the complexity and computational resources could increase. The estimation of the color of the pixel depends on the temporal window size used. For reducing the computational complexity, the estimation is approximated with recursive algorithms based on time differences, *i.e.*, Kalman recursive filters [19], or parameter estimation using Expectation Maximization algorithm [20].

This work assumes that motion objects and background areas can be represented as independent image components *U*_1_,*U*_2_, . . .. The most significant component represents the background image, and the rest of the components represent moving objects, and the less significant may represent noise motion. But, these images are unfortunately, unknown. Using *E*(ϒ) and a set of images ϒ, the estimation of the matrix *W* is performed. This matrix unmixes into a set of images *U*_1_,*U*_2_, . . ., which represent the independent components of fixed and moving objects. Namely, for a particular position **x** we have,

(1) Φ(x)=WΩ(x)

where the *W* matrix separates the mixed color components in Ω(**x**) = [*I*_1_(**x**), *I*_2_(**x**), . . . , *I_n_*(**x**),*E*(ϒ(**x**))]; Φ(**x**) represents the unmixed independent components Φ(**x**) = [*U*_1_(**x**),*U*_2_(**x**), . . . ,*U_n_*(**x**),*U_n_*_+1_(**x**)]. The estimation of *W* needs to express each image in ϒ as a vector. Then, this transformation is performed with *v*(*I_i_*) transformation. This transformation catches spatial information of texture and luminance conditions, expressing any image *I_i_* as a vector form **I***_i_*. The transformation *v*(*I_i_*) is useful if we need to reduce the computer complexity when images are too big, or when there are some restrictions of zones scene. Additionally, the transformation *v*(*I_i_*) implies that the inverse exists *v*^−1^(**I***_i_*), that is, the images encoded as vectors **I***_i_* can be mapped into original images again, preserving the same structure than the original images encoded *I_i_*. For the rest of the document, the bold letter images refer to the vector version resulted from applying *v* transformation. Next, the parameter estimation of the matrix *W* is needed for detecting moving objects from an image sequence. Both processes are discussed in the following sections.

## 3. Estimation of Unmixing Matrix

The matrix *W* is estimated assuming that the pdf of each **U***_i_*(**x**) are both separable and independent from the mixed matrix Ω = [**I**_1_, . . . , **I***_n_*, **I***_n_*_+1_] for all **I***_i_* ∈ ϒ for *n* = 1, . . . ,*n* and **I***_n_*_+1_ = *E*(ϒ), where *E*(ϒ) represents the image estimator and ϒ raw data, *i.e.*, the joint probability of all pdfs are factored as follows

(2) $$p(U_{1},\ldots ,U_{n+1})=p(U_{1})p(U_{2})\;\ldots ,\;p(U_{n+1})$$

where *p*(**U***_i_*) represents the pdf of unmixed images expressed as vector and the independent components are represented in matrix form as Φ = [**U**_1_, . . . ,**U***_n_*,**U***_n_*_+1_]. The parameter estimation is performed by identifying data directions that decrease the Gaussianity of the mixed distributions. In this sense, we can assume that the pdf of the moving objects **u***_i_* has a non-Gaussian distribution. The background distribution behaves mainly as a Gaussian, and it does not affect the component separation process, whenever the other components become non-Gaussian. This assumption establishes the minimum criterion to separate the independent components from the mixed data. In case that all pdfs have a Gaussians distribution, the mixed distribution is symmetrical and is not possible to separate it, *i.e.*, if objects with motion have the same pdf as background, they could have not been identified.

### 3.1. Image Preprocessing

In a preprocessing step, all mixed images **I***_i_* are centered subtracting the mean value *m*(**I***_i_*) so as to make a zero-mean variable. After, each **I***_i_* is uncorrelated with a linear transformation for which its components are equal to one, as follows,

(3) $${\tilde{I}}_{i}={S\Sigma }^{-1/2}S^{T}I_{i}$$

where **Ĩ***_i_* denotes uncorrelated version of **I***_i_*; Σ and **S** result from factorizing covariance matrix $E\left\{I_{i}I_{i}^{T}\right\}={S\Sigma S}^{T}$. Finally, Σ^−1/2^ is computed by a simple component-wised operation.

### 3.2. Measure of Gaussianity

Then, since data are uncorrelated, the weight parameters of *W* = [**w**_1_, **w**_2_, . . . , **w***_n_*]*^T^* are estimated for each row **w***_i_* such that the projection **w_i_***^T^***Ĩ** maximizes the non-Gaussianity. In this sense, the non-Gaussianity degree is measured with an approximation of neg-entropy [14]. The advantages to use neg-entropy instead of kurtosis, for instance, are that is well justified in statistical theory and in some sense, neg-entropy is the optimal estimator of non-Gaussianity as far as statistical properties are concerned. Therefore, a simpler approximation of neg-entropy could be estimated as follows,

(4) $$J(y)\propto {\left[E\{G(y)\}-E\{G(v)\}\right]}^{2}$$

where *G* is commonly a non-quadratic function. The election of any approximation of *G* depends of the scene conditions and the behavior of raw data; however, there are some common approximations of neg-entropy that are shown in Table 2.

### 3.3. Parameter Estimation

At this point, the task to estimate the *W* weight values, is considered as an optimization problem. In this sense, using an approach based on Newton-Raphson like [16], an optimization procedure based on fast ICA [14] is performed to estimate each of the **w***_i_* weight vector parameters in *W* as follows:

1. For each **w***_i_* in *W*
  - Choose initial (*i.e.*, random) values weight for **w***_i_*.
  - Let $w_{i}^{+}=E\{{\tilde{I}}_{i}g(w_{i}^{T}{\tilde{I}}_{i}))\}-E\{g^{\prime }(w_{i}^{T}{\tilde{I}}_{i})\}w$.
  - Let $w_{i}^{+}=\frac{w_{i}^{+}}{\left\|{\tilde{I}}_{i}^{+}\right\|}$.
  - If convergence is not achieved, go back to 1 (a).
2. Continue with the next **w***_i_*

Each internal iteration only estimates one **w***_i_* vector. Then, the algorithm is repeated *n*+1 times, once by each image analyzed in Ω. In [21] is shown a parallel approach to this algorithm, with the advantage of time reduction, against of a considerable reduction in the precision level [22].

### 3.4. The Background Gaussianity and Independent Component Separability

**Figure 1.**
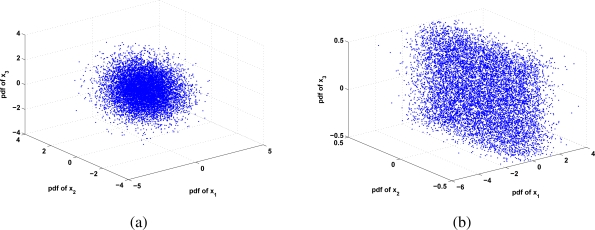
Data distribution for 3 variables (a) when data have a Gaussian Distribution there is not enough information to estimate each independent component (the mixed distribution is totally symmetrical); (b) when data have mixture of non-Gaussian or some of the independent components are Gaussian, it is possible to identify other non-Gaussian components (there are some vortex that define maximums).

The matrix *W* is a linear transformation that separates each non-Gaussian pdf contained in an *I_i_*(**x**) image. But, Stauffer and Grimson [3] have proved experimentally that the background can be modeled as a Gaussian distribution. This implies that there is always one component that is Gaussian. The symmetrical morphology of a Gaussian distribution may affect the optimization steps 1(b) and 1(c), which assume non-Gaussian distribution over each component. In the practice, this constraint can be relaxed, considering the effects that this implies, *i.e.*, if we had all the components distributed as a Gaussian, these could be represented as a hipper ball; which we have not gotten enough geometrical information to separate each independent component (because they are totally symmetrical and they do not have any maximum). But when one or some components are Gaussians, the non-Gaussian components add vertexes (*i.e.*, maximums), which are used to estimate each independent component. Figure 1 illustrates the space conformed by three different pdfs; whenever they follow a Gaussian distribution (Figure 1a) there is not enough information for unmixing, instead, when only one has a Gaussian distribution, the others provide geometrical information for unmixing the space. As it is appreciated, the background Gaussianity does not affect the unmixed process at the parameter estimation process, whenever the pdfs of moving objects have non-Gaussian distribution. This situation may be appreciated as a limitation, but in practice, the majority of moving objects, in short time stamps, does not follow a Gaussian distribution, thus being the only background component considered having Gaussian distribution.

## 4. Motion Detection

The data in Equations 1 and 2 are uncorrelated and normalized, being impossible to rank or define an order over all the estimated components. However, the values contained at *W* provide information about the amount of information over the estimated components. The matrix *W* represents the linear transformation that separates the mixed images as independent components, the inverse of the transformation *W*^−1^ mixes up again the sources *U*(**x**). The linearity of each component expressed in *W* is used to define the importance of each component and, as a consequence, the detection of background, foreground and noise components. The difference between using a PCA [8] approach instead of an ICA, consists in the extra constraint added to the component estimation; *i.e.*, the components must be orthogonal and uncorrelated at same time. Next, from analysis of the singular values of *W*^−1^, the most significant singular value component represents the background data, under the assumption that fixed areas are proportionally greater than moving areas in scene. The next most significant singular values (except for the first one) correspond to moving components, and the last components, represent noise motion.

Then, to separate the first (background component) and last components (noise components) on *W*^−1^ matrix, the singular value decomposition is applied erasing the eigenvalues that correspond to the most significant component and the less significant components. Rebuilding the matrix *W*^−1^ without the first one and the last eigenvalues, the components Ω* are estimated as follows

(5) $$\Omega_{i}^{*}=W{*}^{-1}\Phi =(S\Sigma *D^{T})\Phi$$

where *W*^−1^ = *S*Σ*D^T^*; Σ* = Σ without the most significant and the less significant components; *i.e.*, $\sum_{11}^{*}=0$ and Σ*_ii_* = 0 for {*i*, *i*+1, . . . ,*n*}, which preserves the same information except for the background and noise information; and W*^−1^ = *S*Σ*D^T^. The data, in the reconstructed image Ω*, contain only data corresponding to the moving objects of each independent component **U***_i_* contained in Φ. Next, it is needed to binarize each one of $I_{i}^{*}=v^{-1}(I_{i}^{*})$ that conforms $\Omega *={\left[I_{1}^{*T},I_{2}^{*T},\ldots ,I_{n}^{*T},I_{n+1}^{*T}\right]}^{T}$ for finding out moving objects.

Each particular image $I_{i}^{*}$ does not contain background data and noise data; *i.e.*, the effect of putting Σ_11_ = 0 and some Σ*_ii_* = 0 consists in that the information over each component has been discarded, resulting in spurious data around zero value. In fact, the background corresponds to the majority of the area, and it is represented by the pdf global maximum, and the moving objects by the rest of the pdfs local maximum. The values, that belong to the biggest maximum, are labeled with zero value (which is located around zero value), and rest to one. The resulting binary map represents moving objects as follows

(6) $$B(x)=\{\begin{array}{ll} 0 & \text{if}\;I_{i}^{*}(x)\in \;\text{Background}\\ 1 & \text{other case} \end{array}$$

where the labeling process is achieved via a k-means’s variation [23], which group all the pixels in two categories; background and foreground.

**Figure 2.**
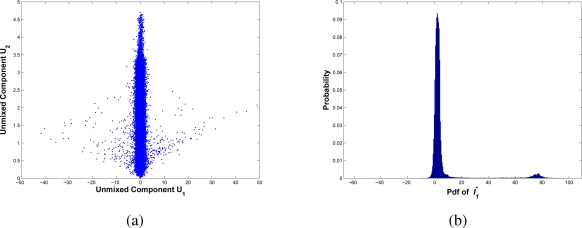
Foreground detection from independent components using the estimation *E*(ϒ) and current image *I_t_*. In (a) the unmixed space as orthogonal components resulted from the pdfs of **U**_1_ and **U**_2_; the main component represents background information (vertical component) and the second component represents foreground objects; In (b) the pdf of estimated $I_{i}^{*}$ after removing background data. The global maxima correspond to background data, and second maxima (right side) correspond to objects with motion.

As an example, using the estimated image *E*(ϒ) and the current image *I_t_*, Figure 2a shows the distribution of independent unmixed components **U**_1_ and **U**_2_, after estimating the unmix matrix *W*. The main component represents the background information (vertical component), and lower components represent the moving objects information (horizontal component). Then, the reconstructed $I_{t}^{*}$ is estimated from the mixing matrix W*^−1^. The pdf of $I_{t}^{*}$ is presented in Figure 2b. The data that surround the zero value correspond to the background; the rest corresponds to the moving components. Finally, the classification task consists on grouping all the elements around zero labeling them as background, and the rest as moving objects.

The motion detection process is invariant to the global luminance variations and diffuse lights. The global luminance variations are tolerated, in sense, they represent the displacement of the pdf along its range. As a consequence, the uncorrelated process always centers and normalizes raw data, being not affected thus by the displacements on range. The diffuse lights affect locally the raw data, but do not affect considerably the pdf distribution of the pixel. Then, this does not have a significant effect in the independent component separation process. The luminance invariants make feasible that this approach supports blur shadows, whenever the pdf morphology is not affected (experimental evidence is presented in §5). Moreover, motion detection may be degenerated when the parameter estimation does not converge, avoiding the correct detection. This happens when both foreground and background have Gaussian distribution, or foreground raw data has not enough evidence to, numerically, estimate its pdf.

As a complement, the component estimation of moving objects could be affected with noise. An additional step consists in applying a connectivity analysis. It can be performed with a morphological operator *P* applied to *B*(**x**) [24], where an useful morphological filter is the opening by reconstruction, which removes low connected areas. The motion areas may be deformed, because, these operators assume that the structural element is not affected by the current image camera projection; *i.e.*, the background is fronto-parallel to the camera view. The opening by reconstruction filter removes elements low-connected than the structural element λ, resulting in a better connected map *B*. Then, the morphological filtering process of the binary map is denoted as follows

(7) $$\tilde{B}=\tilde{\gamma }(B)=\underset{n\to \infty }{\text{lim}}\delta_{B}^{n}(\varepsilon_{\mu }(B))$$

The filtered motion map *B̃* enhances the motion zones discarding additive noise that could affect the process.

## 5. Experiments and Results

In this section we present an experimental model to test our approach and some implementation issues. The experimental model matches the performance of motion detection between MOG [3] and our approach, against a ground truth based on PETS database [17, 18]. The implementation issues point out some useful remarks to reduce the complexity of motion detection.

### 5.1. Implementation Issues

To reduce the computational complexity of the algorithm, we should take the following modification to the approach. The *E*(ϒ) operator should be based on a recursive efficient estimator to compute the expected value [19, 20]. But, we must consider that a simple model could affect the accuracy of the *E*(ϒ) estimator. Additionally, the *v*(*I_i_*) function should sample each image, for avoiding the use of Gaussians filters, which in certain conditions are equivalent [25], but the sampling process reduces the computational resources needed. The number of independent components to be estimated in Equation (1) should be reduced to only the image estimator *E*(ϒ) and the last image acquired. This makes that the unmixed matrix *W* becomes to a 2 × 2 dimension. Now, the second eigenvalue of Σ* in Equation (5) represents the amount of information of moving objects and noise motion, being a necessary thresholding step over a second eigenvalue to detect object motion and noise motion.

### 5.2. Experimental Model

A quantitative process to measure the reliability of a background approach is not an easy task. In background model context, some works like [26] and [27] propose methods for quantifying the degree of success of different background approaches. Moreover, many times, background models are a dependable application, making these proposals not completely general. In this sense, we introduce a quantitative analysis based on the comparison of a well accepted background method as a reference and a set of sequences of images as motion ground truth. Both provided a relative point of comparison to evaluate the effectiveness and accuracy of motion detection in different scenarios.

**Figure 3.**
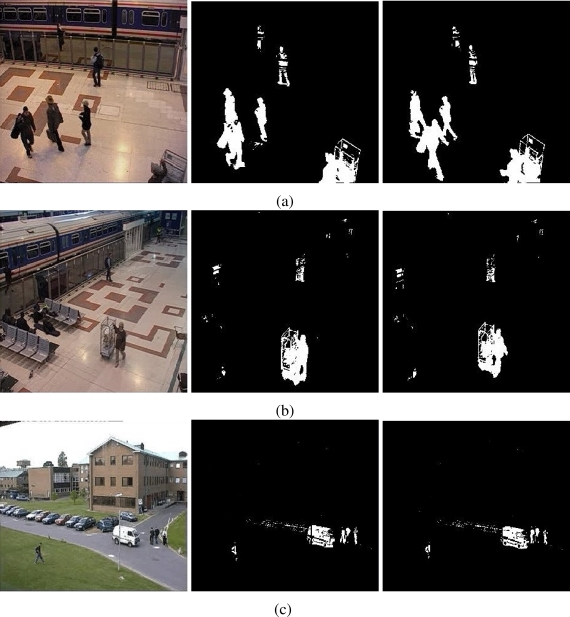
Foreground detection from image sequences of the PETS database [17, 18] using the proposal. The sequences in (a) and (b) correspond to cameras monitoring a train stop; the sequence in (c) corresponds to a car parking. The foreground detection is performed without spatial filter. The level of resolution is higher and soft shadows are discarded automatically.

A performance quantitative measure of our background model consists in comparing motion detection efficiency between our approach and a reference model. The MOG approach [3] is used as a reference model. This approach is a well accepted method and it generally offers good results. To measure the algorithm performance, a motion ground truth is introduced, which is conformed by several sequences of images taken from PETS database [17, 18]. PETS databases are used as a reference for evaluating surveillance algorithms. The sequences of images used are taken from 2001 and 2007 PETS databases, which include indoors/outdoors scenarios. The motion ground truth is performed by hand-selecting motion zones. Only the objects displaced are considered as motion zones, discarding reflections, noise and shadows. We use, as ground truth, three different sequences (see Figure 3). Table 3 summarizes the principal information of sequences used as ground truth.

We introduce three different error measures to compare the two approaches against ground truth data. These errors are expressed in pixels. The first one quantifies the degree of modeling between the foreground estimated map and its ground truth map. This error is well known as BIAS error [28], which, in our context, consists in the arithmetical summation of a binary image difference between motion detection performed by the background approach and its ground truth. This measure provides information about the under/over modeled of motion scenario. Formally, this measure is defined as

(8) $$\varepsilon_{b}=\sum_{x\in B}B(x)-I_{\mathrm{gt}}(x)$$

where *B*(**x**) is the foreground estimated map and *I_gt_*(**x**) is the ground truth map. The second measure, represents the number of pixels labeled as motion zones that correspond to free movement zones. It is commonly named as a false-positive error [29] and it is defined as the complement of a *I_gt_*(**x**) map multiplied point to point with a current motion map *B*(**x**). The error measures near to zero, meaning that the background approach discards efficiently the free movement zones, avoiding thus, the addition of motion noise at *B*(**x**) map. Formally, this error is denoted as follows,

(9) $$\varepsilon_{\mathrm{fp}}=\sum_{x\in B}\bar{B(x)}*I_{\mathrm{gt}}(x)$$

where $\bar{B(x)}$ is the complement of the map of motion detected *B*(**x**) and the operator * is the single multiplication. The third measure represents the number of pixels labeled as free motion zones that correspond to motion zones. This error is named as a false-negative error and represents the capabilities of our approach to model correctly motion zones. Error measures near to zero, mean that all objects with motion have been adequately detected. Consequently, error measures greater than zero, mean that the background approach does not detect efficiently the motion areas. This error measure is defined as follows

(10) $$\varepsilon_{\mathrm{fn}}=\sum_{x\in B}B(x)*\bar{I_{\mathrm{gt}}(x)}$$

where $\bar{I_{\mathrm{gt}}(x)}$ is the complement of ground truth map and * operator is single multiplication.

Next, the approach is tested with a set of sequences taken from different outdoor/indoor scenarios. These scenarios show different common situations in surveillance systems like luminance disturbance, shadows, reflection, *etc.*

Then, the work is compared with the PCA approach (see Appendix A) for segmenting the background from the foreground. Finally, it is shown the results for applying a morphological analysis to improve the quality of the motion map.

### 5.3. Results and Discussions

The proposal and MOG approach are tested as we described above. The proposal was implemented with only two components (the estimator of images *E*(ϒ) and the last frame acquired), the function *v*(*I*) is defined as the concatenation of odd columns conformed by odd pixel positions. The threshold to identify moving objects and noise is defined to 0.05. The operator *E*(ϒ) is defined as an average operator to estimate background information with a window of 100 frames. The MOG approach was implemented using a ρ = 0.005, and 3σ as belonging criterion. The background initialization was performed with 100 frames. All tests are performed without applying any connectivity criterion.

**Figure 4.**
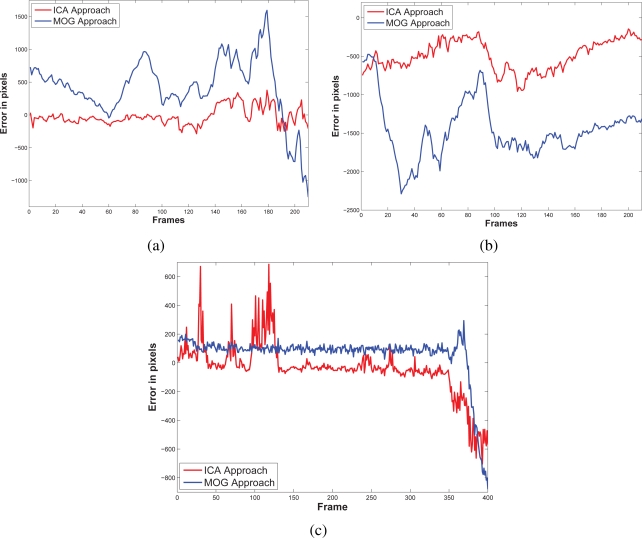
Bias error resulted to compare ground truth against our approach and the MOG approach. In (a) it is shown the bias error of the first scenario that corresponds to a train station. In (b), the same train station is monitored from other perspective, where the reflection, the shadows, and the perspective make more complicated the motion detection. In (c) the error bias at an outdoors scenario; the noise in the sequence affects negatively the motion detection. The proposed approach generally offers a smaller bias error than the MOG approach.

**Figure 5.**
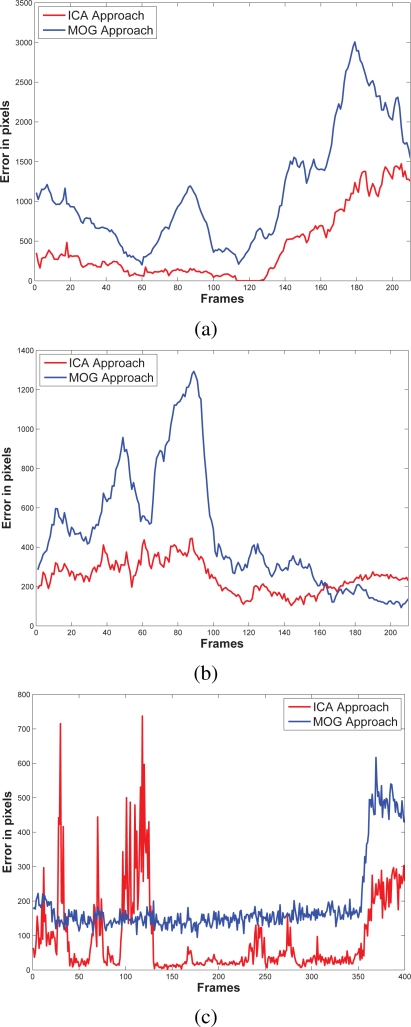
False-positive errors resulted after comparing ground truth against our approach and the MOG approach. In (a), the first train station scenario, where the MOG approach is more sensitive to introduce noise; on the other hand, the proposal adds less error of motion. In (b) the second scenario of the train station at a different perspective, the behavior is similar, except for the end, our proposal has a significative major error bias. In outdoor scenarios (c), the MOG is more sensitive to luminance variations than our proposal.

**Figure 6.**
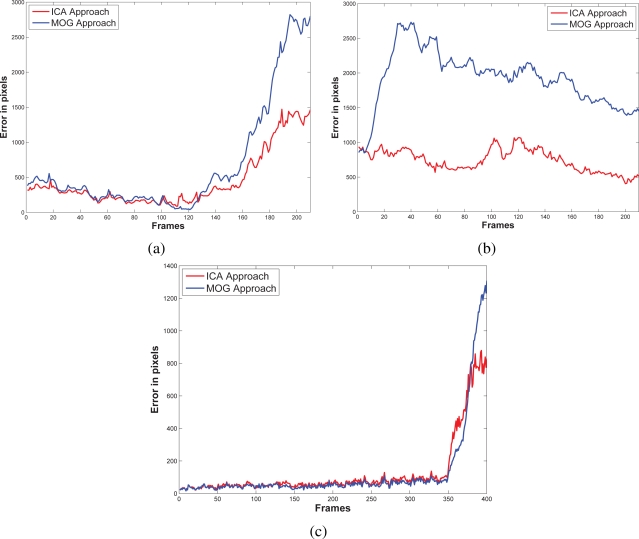
False-negative errors resulted after comparing ground truth against the proposal and the MOG approach. In the first train station sequence, (a) both errors are similar except for the end of sequence, where our approach has better accuracy for segmenting moving objects. In second train station sequence (b), the MOG approach is more sensitive to people shadows and reflection. Instead, the proposal always has better accuracy for detecting object moving. Finally, in (c) both measures are similar, except at the end of sequence, where our approach offers better accuracy than the MOG approach.

**Figure 7.**
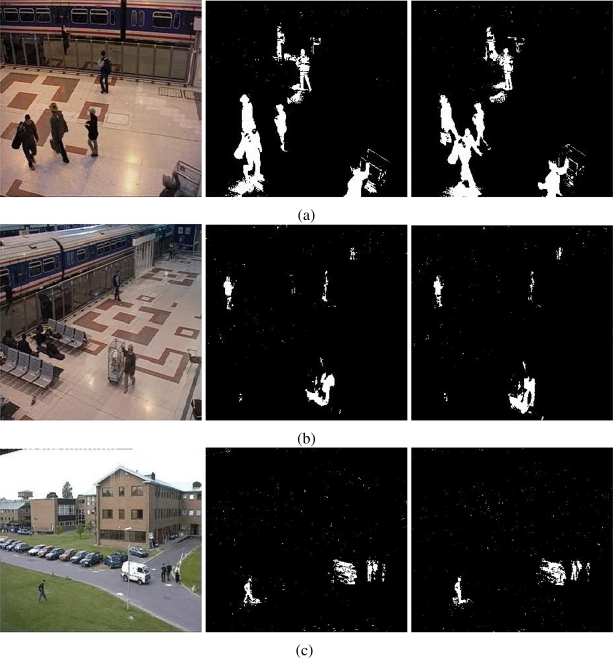
Foreground detection from images sequences of the PETS database [17, 18] using the MOG approach[3]. The sequences in (a) and (b) correspond to cameras monitoring a train stop; the sequence in (c) corresponds to a car parking. The objects detected are not well defined, and in several frames, some objects are discarded (especially in thin object structures). The motion detection is sensitive to shadows and images are pruned with noise.

The results of testing the proposal and the MOG approach are showed in Figures 4–6. Additionally, Figures 3 and 7 show some frame motion detected with both approaches. As it is appreciated, in Figure 4, the MOG approach is generally more sensitive to changing light conditions in the scene; on the other hand, the proposed approach becomes more stable in tested scenes. The bias error is greater with a MOG approach than the proposal, *i.e.*, the proposal detects better objects motion in the sequences of images.

The false-positive error quantifies when the approach adds noise from motion to the motion detection map. In this sense, we appreciate in Figure 5 that the MOG approach detects false motion zones. The false motion zones are difficult to deal with, and several times, it would be confusing with the motion objects, degrading the post analysis stages in vision systems. Mainly, the noise is caused by the sudden reflections and the shadows caused by the objects moving, like people walking down (Figure 7a), in MOG approach; but in the proposal, the error measure is small, behaving more efficiently than the MOG approach (Figure 3a). In outdoor scene, MOG error remains constant and usually greater than the proposed approach. Thus, the MOG approach produces binary maps of motion affected by noise, whereas the proposal produces binary maps of motion more clearly, without applying any connectivity analysis. The sudden noise peaks are caused particularly when objects are too small (a deep discussion is made in the following paragraphs).

On the other hand, the false-negative error quantifies the accuracy to detect foreground areas. In this sense, we appreciate that the MOG approach usually detects correctly objects with motion. However, when foreground has a high similarity degree with background or it is constituted by big flat surfaces, the MOG approach only detects foreground contours. This fact causes a high false-negative error with the MOG approach (see Figure 6a–c). The proposal estimates and separates the mixed pdfs that conforms background and foreground zones. Consequently, it produces a better motion segmentation, and a small false-negative error. In Figure 6a both approaches behave too similar. The small variations are caused when skin zones belonging to people walking down are too similar with background. In Figure 6b, the camera perspective and the reflections difficult the process of foreground detections, causing that people walking down are not detected. Finally, in outdoor scenarios, the small objects are detected efficiently with MOG; however, big objects with slow motion are broken (Figure 7c). But our approach segment with fewer noise degree, and the slow movement objects are better segmented (Figure 3c). Consequently, the error level is similar in both cases, except for the end of the sequence that corresponds to a vehicle with slow motion. Visually, the results can be appreciated in Figures 3 and 7.

**Figure 8.**
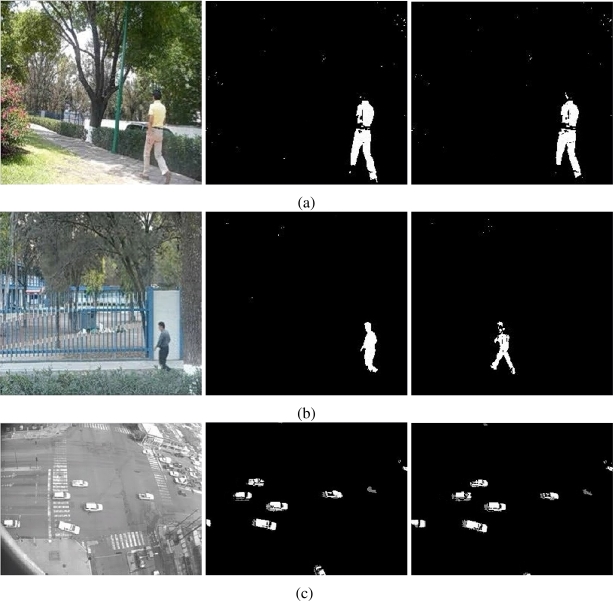
Foreground detection from (a) a camera monitoring a public garden, (b) a camera monitoring a people lane, (c) a camera monitoring an intersection. The first two images show several leafs moving caused by wind and the scene is continuously changing. The third one, shows cloud shadows causing changes of luminance conditions.

**Figure 9.**
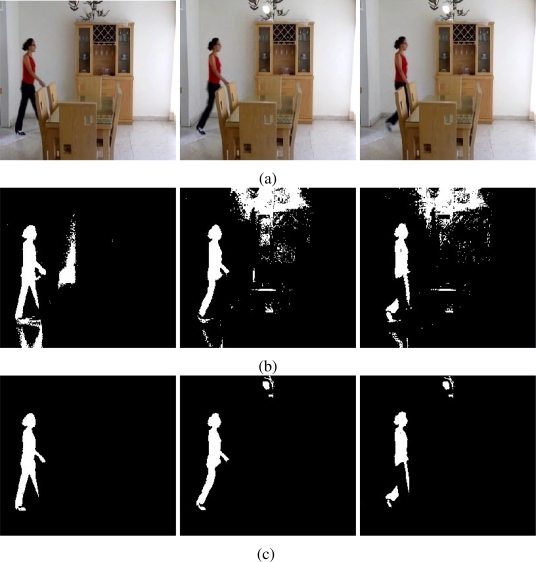
Foreground detection at a scene with sudden luminance change. In (a), we present a room, where light is turning on, the floor shows reflections and the wall shows soft shadows. (b) When the MOG approach is applied, the reflections and the shadows are detected as moving objects. (c) Instead, the proposal is capable to detect better the human silhouette without the introduction of moving noise, even the light condition has changed.

Additionally, the proposal is tested with different image sequences from distinct scenarios. The sequences of images correspond to outdoor scenarios, where the luminance conditions are changeable. The sequences of images were taken from gardens with people walking down and at a vehicular intersection. Figure 8 shows frames belonging to different outdoor sequences. The results show that the proposal is capable to identify foreground objects efficiently. Figure 8a,b shows people walking down in outdoor gardens. Both experiments use the median operator as estimator *E*(ϒ) and $\frac{1}{a_{1}}$ log cosh *a*_1_*u* with *a*_1_ = 1 to estimate neg-entropy. The images present few noise effects caused by the reflectance and luminance conditions of different objects and materials. In Figure 8c, the object motion corresponds to vehicles at intersections. The scene is affected by vehicle reflections and cloud shadows. The foreground zones are detected efficiently with a weight-average estimator for *E*(ϒ) and a Gaussian for neg-entropy. Finally, in Figure 9, it is presented a scenario with sudden luminance changes. Figure 9a shows some frames used to illustrate the environment conditions. The floor and the wall are affected by soft shadows caused by a woman walking down and the sudden light turned on. Figure 9b shows the results with the MOG approach. The soft shadows, reflections and sudden luminance changes affect negatively the foreground detection. However, it is appreciated that the approach is capable to discard the majority of the soft shadows and reflections. As an example, when light is turned on, foreground detection is performed adequately.

**Figure 10.**
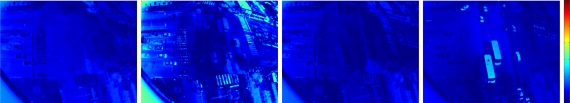
Images differences amongst the components estimated with the proposal and the PCA estimation. The main differences correspond to the areas affected by several lights, as the roof of the vehicles and the buildings, and, in minor sense, the global lights effects over the scenario.

The ICA approach can be considered as an extension of the PCA approach (see Appendix A), in this sense, an approximation of the motion detection process could be performed using PCA approach. The results are similar when the second order independence is equivalent; in other cases, they are different. This is, when in the PCA approach, the principal components are both, independent and uncorrelated, the PCA is equivalent to the ICA. In the practice, both are equivalent when the pixels suffer global effects.But, when scenario have several luminance sources, there are some considerable differences. To illustrate it, Figure 10 shows the differences of I* and the second principal component estimated with the PCA approach, using the the sequence of the intersection. The images are in pseudo-color where dark zones represent small differences, and red color, considerable differences. The main differences are detected in zones with shadows and reflecting materials. The proposal identifies the objects better as the local distribution of pixels values must be independent and uncorrelated of the estimation *E*(ϒ); in contrary, the PCA approach is enough that data are independent. In the practice, the differences depend of the optimization process to estimate *W* and the approximation of neg-entropy used. But, the results are always better in the proposal, given that it adds the constraint of uncorrelation that the PCA does not consider.

**Figure 11.**
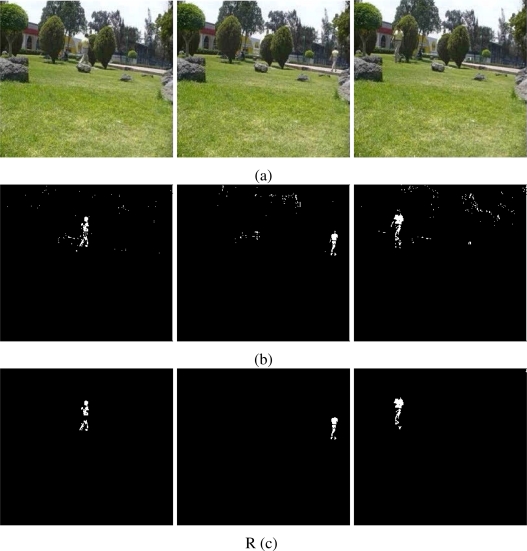
The enhancement of binary motion maps. The motion objects are better defined, and the noise effects are discarded. In (a) the original frames; in (b) the estimation of *B*(**x**), and in (c) the enhancement map *B̃*(**x**).

As additional results, the binary motion map *B*(**x**) is improved with a opening by reconstruction filter. This filter helps out to clarify the motion zones and eliminates noise classification regions. Figure 11 shows some frames acquired from a public garden, where luminance conditions are changeable. The motion detection is performed, but it is affected with noise. The morphological filter eliminates those regions low-connected. The resulting image offers a better definition of the motion objects. However the filters must be used carefully, because they require more computational resources and affects the frame rate specially in real time systems.

The proposal method works efficiently to detect foreground objects when data information that represents the foreground is separable from the background. The proposal method offers a numerical approximation, and in practice, it needs to consider some points that could affect the foreground detection. The level of accuracy depends of the convergence criterion of the algorithm showed in §3.3, the number of components that are considered as noise data in Σ*, and the amount of information to estimate each independent component; consequently, the small foreground zones are difficult to be detected. As an example, we can see in Figures 3 and 6 where a person walking down is not completely detected.

## 6. Conclusions

In this work, we presented a background subtraction approach based on an independent component analysis. This approach exploits the separability and the non-Gaussianity to estimate each of the pdfs. The motion is efficiently detected when raw data are separable. The results show a robust approach at several scenarios. The moving objects are detected with high resolution even when the scenario presents luminance changes or shadows. The implementation issues have been discussed and this approach can be implemented in a real time monitoring system. Finally, this approach has a better accuracy than the MOG approach in the tested scenarios and is superior in scenarios with changing environmental conditions.

## Figures and Tables

**Table 1.** List of some common function estimator *E*(ϒ) for pixel values.

**No.**	**Estimator**
1	*E*(ϒ) = *Ĩ*(**x**) where each $\tilde{I}(x)=\frac{1}{\left\|ϒ\right\|}\sum_{i\in ϒ}I(x)$
2	*E*(ϒ) = *Ĩ*(**x**) where each *Ĩ*(**x**) = median {*I_i_*_∈ϒ_(**x**)}
3	*E*(ϒ) = *μ***_x_** where each *Ĩ*(**x**) ∼ *G*(*μ***_x_**, σ**_x_**)

**Table 2.** Common estimator functions *G* for a neg-entropy approximation.

**No.**	**Function**
1	$G(u)=\frac{1}{a_{1}}\text{log cosh}\;a_{1}u\;\text{for}\;0\leq a_{1}\leq 1$
2	$G(u)=-e^{\frac{-u^{2}}{2}}$
3	*G*(*u*) = *u*^3^

**Table 3.** List of sequences of images used as ground truth. These images present complex scenarios. The first two sequences represent scenarios with shadows caused by different light sources and both color and texture of skin in some frames, that are too similar to background scenario. The third one, represents a scenario with high noise degree, the reflections are caused by the windows and the moving objects are represented by small zones.

**No.**	**Place**	**Source**	**Num. Img.**^1^	bf Num. Train. ^2^
1	Train Station	PETS 2007	300	100
2	Train Station	PETS 2007	300	100
3	Outdoors Park	PETS 2001	500	100

1 Total number of images used.

2 Number of images used to train background model approach.

## A Motion Detection and PCA approach

The PCA approach is used as a subspace projection technique. In PCA, the basis vectors are obtained by solving the algebraic eigenvalue system *R^T^* (**XX***^T^*)*R* = Σ, where *X* is the centered data, *R* is a matrix of eigenvectors, and Σ is the corresponding diagonal matrix of eigenvalues. The projection of the data $C_{n}=R_{n}^{T}X$, from the original *p* dimensional space to a subspace spanned by *n* principal eigenvectors is optimal under the mean squared error; *i.e.*, the projection of **C***_n_* back into the *p* dimensional space, has a minimum reconstruction error. In fact, if *n* is large enough to include all the eigenvectors with non-zero eigenvalues, the projection is lossless.

Thus, the goal in the PCA approach is to minimize the projection error from compressed data, the goal of the ICA approach is to minimize the statistical dependence between the basis vectors; *i.e.*, this can be written as *W***X***^T^* = **U**, where the ICA approach searches for a linear transformation *W* that minimizes the statistical dependence between the rows of **U**, given a training set **X**. Unlike PCA, the basis vectors in ICA are neither orthogonal nor ranked in order. Also, there is no closed form expression to find *W*. This work is based on the FastICA approach [21], which use the neg-entropy to estimate *W*, given that it is invariant to linear transformation [15]. This is, the estimation of *W* minimizes the mutual information, resulting roughly equivalent to find the directions in which the neg-entropy is maximized. This formulation shows explicitly the connection between the ICA and projection pursuit. In fact, finding a single direction that maximizes neg-entropy is a form of projection pursuit, and could also be interpreted as estimation of a single independent component. Thus ICA is an extension of PCA approach with the adds of the imposing independence up to the second order and, the definition of directions that are orthogonal as discussed in [15].

The Motion Detection process uses the middle-significant eigenvalues of the mixing matrix *W*^−1^; *i.e.*, there is a dimensionality reduction. However, the reduction is performed in the unmixed data **U**. Then, the dimensionality reduction on this matrix could result equivalent to PCA, when the second order independence are equivalent to the obtained with the PCA estimation.
